# Supplementary material for: Impact of Music Interventions on Depression in Care Home Residents with Dementia: UK Results from Music Interventions for Depression and Dementia in Elderly Care RCT
Source: Geriatrics (Basel). 2025 Dec 15;10(6):166. doi: 10.3390/geriatrics10060166 (PMC12732711; doi:10.3390/geriatrics10060166)
Supplement: Supplementary file 1 [file geriatrics-10-00166-s001.zip › Figure S1_Consort Diagram.pptx]

## Slide 1
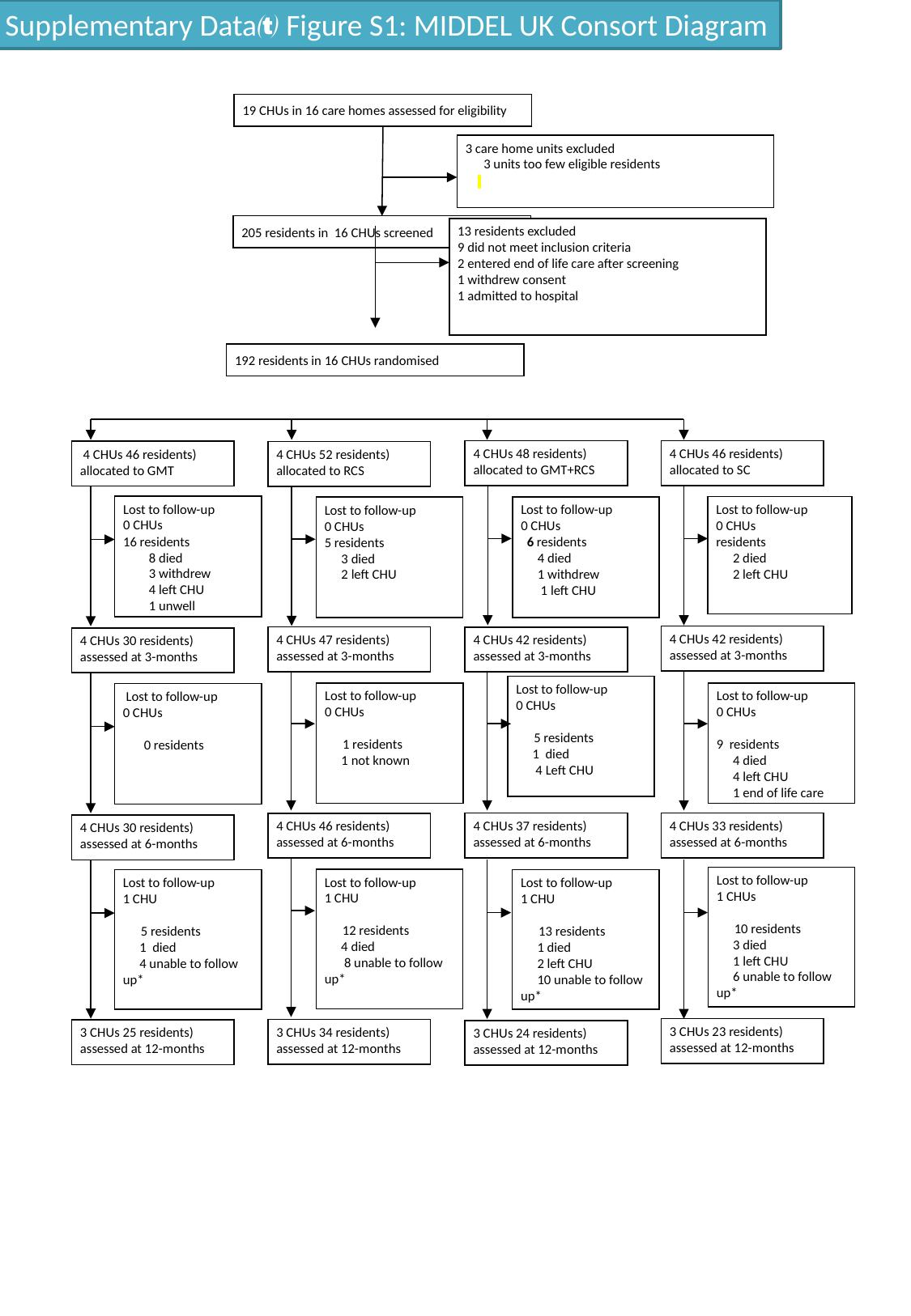

Supplementary Data Figure S1: MIDDEL UK Consort Diagram
19 CHUs in 16 care homes assessed for eligibility
3 care home units excluded
 3 units too few eligible residents
205 residents in 16 CHUs screened
4 CHUs 48 residents) allocated to GMT+RCS
4 CHUs 46 residents) allocated to SC
 4 CHUs 46 residents) allocated to GMT
4 CHUs 52 residents) allocated to RCS
Lost to follow-up
0 CHUs
16 residents
 8 died
 3 withdrew
 4 left CHU
 1 unwell
Lost to follow-up
0 CHUs
residents
2 died
2 left CHU
Lost to follow-up
0 CHUs
 6 residents
4 died
1 withdrew
 1 left CHU
Lost to follow-up
0 CHUs
5 residents
3 died
2 left CHU
4 CHUs 42 residents) assessed at 3-months
4 CHUs 47 residents) assessed at 3-months
4 CHUs 42 residents) assessed at 3-months
4 CHUs 30 residents) assessed at 3-months
Lost to follow-up
0 CHUs
5 residents
1 died
 4 Left CHU
Lost to follow-up
0 CHUs
1 residents
1 not known
Lost to follow-up
0 CHUs
9 residents
4 died
4 left CHU
1 end of life care
 Lost to follow-up
0 CHUs
 0 residents
4 CHUs 33 residents) assessed at 6-months
4 CHUs 37 residents) assessed at 6-months
4 CHUs 46 residents) assessed at 6-months
4 CHUs 30 residents) assessed at 6-months
Lost to follow-up
1 CHUs
10 residents
3 died
1 left CHU
6 unable to follow up*
Lost to follow-up
1 CHU
12 residents
4 died
 8 unable to follow up*
Lost to follow-up
1 CHU
5 residents
1 died
4 unable to follow up*
Lost to follow-up
1 CHU
13 residents
1 died
2 left CHU
10 unable to follow up*
3 CHUs 23 residents) assessed at 12-months
3 CHUs 34 residents) assessed at 12-months
3 CHUs 25 residents) assessed at 12-months
3 CHUs 24 residents) assessed at 12-months
13 residents excluded
9 did not meet inclusion criteria
2 entered end of life care after screening
1 withdrew consent
1 admitted to hospital
192 residents in 16 CHUs randomised
