# Supplementary material for: Impact of Music Interventions on Depression in Care Home Residents with Dementia: UK Results from Music Interventions for Depression and Dementia in Elderly Care RCT
Source: Geriatrics (Basel). 2025 Dec 15;10(6):166. doi: 10.3390/geriatrics10060166 (PMC12732711; doi:10.3390/geriatrics10060166)
Supplement: Supplementary file 1 [file geriatrics-10-00166-s001.zip › Table S1 PPA analysis.pdf]

Table S1: Per-protocol Analysis

**Table S1 a: MADRS**	**2-way-time interaction**		**3-way interaction**	
	**B (95%CI)**	**p-value**	**B (95%CI)**	**p-value**
Time effect[1]				
3 months	-7.8 (-10.4, -5.2)	**<0.001**[2]	-7.9 (-10.6, -5.2)	**<0.001**
6 months	-9.4 (-12.1, -6.7)	**<0.001**	-9.8 (-12.6, -6.9)	**<0.001**
Main effects				
RCS	3.5 (0.4, 6.6)	0.032	3 (-0.5, 6.5)	0.102
GMT	-4.7 (-8.3, -1.1)	**0.012**	-5.5 (-9.9, -1.1)	**0.018**
Interaction time*RCS				
3 months * RCS	-2.5 (-6.2, 1.2)	0.195	-2.2 (-6.4, 2)	0.311
6 months * RCS	1.5 (-2.3, 5.3)	0.44	2.3 (-2, 6.6)	0.303
Interaction time*GMT				
3 months * GMT	3 (-1.2, 7.2)	0.173	3.4 (-1.8, 8.6)	0.205
6 months * GMT	5.8 (1.5, 10)	**0.01**	7 (1.7, 12.2)	**0.012**
Interaction RCS*GMT			2.4 (-5.3, 10)	0.552
3-ways interactions				
3 months * RCS * GMT			-1.3 (-10.3, 7.6)	0.775
6 months * RCS * GMT			-3.5 (-12.5, 5.5)	0.453
**Table S1b: SIB-8**				
Time effect[1]				
3 months	0.2 (-0.7, 1.2)	0.676	0.2 (-0.8, 1.2)	0.712
6 months	0.8 (-0.3, 1.8)	0.15	1 (-0.1, 2.1)	0.084
Main effects				
RCS	0.5 (-1.9, 2.8)	0.692	-0.2 (-2.8, 2.4)	0.893
GMT	2.1 (-0.6, 4.8)	0.141	1 (-2.3, 4.3)	0.547
Interaction time*RCS				
3 months * RCS	-0.6 (-2, 0.7)	0.385	-0.6 (-2.1, 1)	0.474
6 months * RCS	-2.7 (-4.2, -1.1)	**<0.001**	-3.2 (-4.9, -1.4)	**<0.001**
Interaction time*GMT				
3 months * GMT	-0.1 (-1.6, 1.5)	0.943	0 (-1.9, 1.9)	0.993
6 months * GMT	0.4 (-1.3, 2.1)	0.642	-0.3 (-2.5, 1.8)	0.756
Interaction RCS*GMT			3.1 (-2.6, 8.8)	0.292
3-ways interactions				
3 months * RCS * GMT			-0.2 (-3.5, 3.1)	0.905
6 months * RCS * GMT			2 (-1.5, 5.6)	0.271
**Table S1c: Neuropsychiatric Inventory: Symptom Severity**				
Time effect[1]				
3 months	-3.4 (-5.5, -1.2)	**0.003**	-3.1 (-5.3, -0.8)	**0.008**
6 months	-4.3 (-6.5, -2.1)	**<0.001**	-4 (-6.4, -1.7)	**0.001**
Main effects				
RCS	2.9 (0.4, 5.3)	**0.024**	3.7 (0.9, 6.4)	0.011
GMT	-1 (-3.8, 1.8)	0.477	0.2 (-3.2, 3.7)	0.905
Interaction time*RCS				
3 months * RCS	-3.4 (-6.5, -0.3)	0.032	-4.2 (-7.7, -0.7)	**0.022**
6 months * RCS	1.2 (-2, 4.3)	0.474	0.4 (-3.2, 4)	0.821
Interaction time*GMT				
3 months * GMT	1.5 (-2, 5)	0.414	0.3 (-4, 4.7)	0.883
6 months * GMT	2.6 (-1, 6.2)	0.162	1.4 (-3, 5.8)	0.533
Interaction RCS*GMT			-3.7 (-9.7, 2.2)	0.227
3-ways interactions				
3 months * RCS * GMT			3.5 (-4, 10.9)	0.369
6 months * RCS * GMT			3.5 (-4, 11)	0.374

[1] Time effect includes control group
[2] Bonferroni correction applied, bringing threshold of statistical significance to $p<0.025$

Table S1d: Neuropsychiatric Inventory – Caregiver Distress

Time effect[1]				
3 months	-4.4 (-7.2, -1.5)	**0.003**	-3.1 (-6, -0.2)	0.04
6 months	-7.1 (-10.1, -4.1)	**<0.001**	-5.4 (-8.5, -2.4)	**<0.001**
Main effects				
RCS	7.1 (3.6, 10.6)	**<0.001**	9.2 (5.3, 13.1)	**<0.001**
GMT	-2.5 (-6.6, 1.6)	0.235	0.9 (-4, 5.7)	0.736
Interaction time*RCS				
3 months * RCS	-8 (-12.2, -3.9)	**<0.001**	-11.2 (-15.7, -6.7)	**<0.001**
6 months * RCS	-3.7 (-8, 0.5)	0.09	-7.6 (-12.2, -3)	**0.002**
Interaction time*GMT				
3 months * GMT	2.9 (-1.9, 7.6)	0.245	-2.1 (-7.7, 3.5)	0.47
6 months * GMT	5 (0.2, 9.8)	0.046	-0.9 (-6.6, 4.8)	0.764
Interaction RCS*GMT			-10 (-18.5, -1.6)	**0.024**
3-ways interactions				
3 months * RCS * GMT			14.7 (5.1, 24.3)	**0.004**
6 months * RCS * GMT			17.1 (7.4, 26.8)	**<0.001**

Table S1e: Quality of Life: EQ-VAS

Time effect[1]				
3 months	8.2 (1.5, 15)	**0.02**	7.3 (0.2, 14.4)	0.048
6 months	7.5 (0.4, 14.5)	0.043	7.7 (0.2, 15.1)	0.05
Main effects				
RCS	-8.5 (-17.1, 0.1)	0.057	-13.6 (-23.1, -4.2)	**0.006**
GMT	9.6 (-0.5, 19.7)	0.066	1.1 (-11.2, 13.4)	0.862
Interaction time*RCS				
3 months * RCS	1.2 (-8.5, 10.9)	0.814	3.6 (-7.4, 14.5)	0.531
6 months * RCS	3.4 (-6.6, 13.3)	0.514	3.3 (-8, 14.7)	0.572
Interaction time*GMT				
3 months * GMT	0.7 (-10.5, 12)	0.899	5 (-9, 18.9)	0.495
6 months * GMT	-1.9 (-13.3, 9.4)	0.745	-1 (-15.2, 13.1)	0.888
Interaction RCS*GMT			24.3 (3.7, 44.9)	0.024
3-ways interactions				
3 months * RCS * GMT			-11.6 (-35, 11.8)	0.342
6 months * RCS * GMT			-2.1 (-25.7, 21.5)	0.864

Table S1f: Quality of Life QOL-AD

Time effect[1]				
3 months	0.3 (-1.7, 2.3)	0.766	0.1 (-2, 2.1)	0.935
6 months	-1.4 (-3.6, 0.7)	0.191	-2.5 (-4.6, -0.3)	0.03
Main effects				
RCS	-1.8 (-4.9, 1.3)	0.259	-3.2 (-6.6, 0.3)	0.076
GMT	2.8 (-0.7, 6.4)	0.126	0.6 (-3.7, 4.9)	0.781
Interaction time*RCS				
3 months * RCS	-0.9 (-4, 2.1)	0.546	-0.4 (-3.7, 2.9)	0.832
6 months * RCS	2.4 (-0.7, 5.4)	0.133	4.8 (1.4, 8.2)	**0.007**
Interaction time*GMT				
3 months * GMT	0.8 (-2.7, 4.3)	0.652	1.7 (-2.4, 5.9)	0.425
6 months * GMT	1.7 (-1.8, 5.2)	0.34	5.5 (1.3, 9.7)	**0.013**
Interaction RCS*GMT			6.6 (-0.9, 14.1)	0.091
3-ways interactions				
3 months * RCS * GMT			-2.7 (-9.9, 4.5)	0.465
6 months * RCS * GMT			-10.9 (-18.2, -3.7)	**0.004**
