# Supplementary material for: Impact of Music Interventions on Depression in Care Home Residents with Dementia: UK Results from Music Interventions for Depression and Dementia in Elderly Care RCT
Source: Geriatrics (Basel). 2025 Dec 15;10(6):166. doi: 10.3390/geriatrics10060166 (PMC12732711; doi:10.3390/geriatrics10060166)
Supplement: Supplementary file 1 [file geriatrics-10-00166-s001.zip › UK GMT Handbook.pdf]

Group Music Therapy: Intervention Handbook

Version 1.0 December 1, 2021

Authors

Professor Felicity Baker, University of Melbourne: felicity.baker@unimelb.edu.au

Professor Christian Gold, UniResearch. Bergen, Norway

Dr Jeanette Tamplin, University of Melbourne

Dr Imogen Clark, University of Melbourne

Dr Claire Lee, University of Melbourne

Funded by the National Health and Medical Research Council, Australia

Adapted by Professor Justine Schneider, University of Nottingham, for MIDDEL UK

Contents

Group Music Therapy: Intervention Handbook 1
- Authors 1
- Overview of intervention 3
- Essential skills 3
- Key responsibilities 3
- Goals of the protocol 4
- Core aims of GMT 4
- Music Therapy Assessment 5
- Other information 5
- GMT format 6
  - Setting up group sessions: preparation 6
  - After the session 6
- Session activities 7
  1. Welcome song and Introduction 7
  2. Song singing and reminiscence discussions 7
  3. Improvisation on instruments 7
  4. Spontaneous or directed movement to music 8
  5. Goodbye song and farewell 8
- Role of Music Therapist facilitators 9
- Professional Supervision and Self-Assessment 10
- GMT Clinical competencies 11
- GMT Clinical scenarios – Problem solving 11
- Test yourself 12
- Appendix 1: Music preference form and additional questionnaire for family members 13
- Appendix 2: Attendance Register to complete at every session 16
- Appendix 3: Covid precautions form to complete at every session 17
- Appendix 4: GMT Fidelity Scale 18

Group size = 8-10 participants

Weeks 1-13, 2 x 45 minute sessions

Weeks 14-26, 1 x 45 minute sessions per week

Essential skills

- Experience working clinically with people living with dementia
- Broad musical skills and repertoire suitable for people living in residential care facilities
- Sound written and communication skills including the ability to communicate with a range of stakeholders including nursing staff, medical staff, families and university staff
- Demonstrated ability to work independently and collaboratively in a team to achieve goals and meet deadlines
- Strong organisational skills

Key responsibilities

- Conducting music therapy sessions with small groups according to the research protocol in multiple facilities involved in the study
- Liaising with nursing and care staff to ensure participants are ready to participate in groups at the scheduled time
- Recording attendance and entering material into databases as required
- Reporting daily on sessions conducted
- Recording session notes
- Video recording sessions as determined by the research team
- Occupational Health and Safety (OH&S) and Environmental Health and Safety (EH&S) responsibilities

Goals of the protocol

The core principle is affect regulation through active, reciprocal music making.

Music making

- facilitates the relationship between the music therapist and the PWD, and between participants in the group.
- takes into account the level of dementia severity and symptoms that can vary from person to person and from session to session.

The core intention of GMT is to meet the psychosocial needs of each person living with dementia, which in turn is thought to reduce depressive symptoms and anxiety and to stimulate overall social and emotional wellbeing

This works in the 'here and now' by responding to immediate emotional expressions, acknowledging them, and validating their emotions by transforming them into meaningful musical expressions for therapeutic gain.

It features concepts of personhood (recognition, trust, respect). Cf McDermott et al. 2014

Core aims of GMT

The core aims of the GMT are:

- to meet the psychosocial needs of PWD
- to empower the PWD with resources that promote affect regulation and attunement
- to foster empathic relationships
- to foster and improve verbal, non-verbal, and musical communications between the PWD and other group members
- to reduce behavioural and psychological symptoms (agitation, apathy, depression etc) through regulation of emotions
- to foster relationship building through musical interactions,
- to target mental stimulation, mood enhancement, reciprocal interaction, and human connection
- to support responses in the here and now
- to provide flexibility to support participation.
- to support identity/history of person.

Music Therapy Assessment

Initial 20-minute assessment with a Music Therapist has the following objectives:

1. Determine what participant's musical preferences are
2. Start building individual rapport
3. Speak with participant's family/carers and test out various songs to see which yield a response.

It is most likely that playing/singing excerpts of songs and observing responses will be the best way to gain insight into a participant's musical preferences.

The therapist will also interview the PWD and/or the family in an effort to determine the musical biography of the participant, their cultural background, history, personal strengths/resources, and disabilities, and any other information that could be useful to bring into music therapy sessions.

Other information

Where possible, the music therapist may also interview care staff and family members. Before undertaking this it is important that the person being interviewed has undergone a formal consent process to participate in the research. Interviews are meant to gain further details including:

- where the PWD was born,
- last school attended,
- main occupation/spouse's main occupation,
- last job, when and why retired
- names of children (if they have any), partner etc

This information may be useful in subsequent music therapy sessions to foster relationships and to support a focus on "who you are." See Appendix 1: Music Preference Form

GMT format

Setting up group sessions: preparation

Tasks to be completed **prior to each session**:

Reminder/confirmation of visit the morning of each session (to be arranged with each residential care home manager) – phone call/email

Prepare device for audiovisual recording: check it is charged up

In collaboration with residential care home staff, set up chairs and place large print lyrics on chairs

Set out percussion instruments, props, and other equipment (eg, bluetooth speakers) to be used during the session

Prepare attendance list for each session

Learn repertoire that is identified in the music therapy assessment as being meaningful to participants

Song selection – sourcing of sheet music / chord charts as required

Environment and equipment suggestions:

Cosy room for up to 12 people (in case visitors come),

Piano or good quality keyboard with functions for transposing, rhythmic loops, changing sounds or guitar and stand

Percussion instruments including djembe drums, bongos, tambourines, and shakers

Bluetooth speaker

After the session

Turn off audio-visual recorder.

Pack up and assist where necessary to take participants back to rooms or general areas.

Check completion of session documentation – see Appendix 2 (Attendance Form) & Appendix 3 (Covid form). Appendix 4 (Fidelity Scale) is completed outside the session.

Check in with Joanne Ablewhite to see if all documentation has been complete and that audio-visual files have been synced to the cloud.

Session activities

1. Welcome song and Introduction

- Choose a welcome song suitable for the group and use this same song each week as a ritual hello song;
- Remind participants who you are and do introductions/re-introductions;
- If applicable, give a short recap of activities of the previous session;
- Tell participants what the layout of the session will be.

2. Song singing and reminiscence discussions

- Singing songs of participant choices (up to one song per participant or where preferences are shared, there may be less) (approximately 15 minutes);
- Engage individuals using eye contact, gestures, adapting vocal style to attune to participants' level of energy, both when singing and talking;
- Engage participants with discussion/reminiscence opportunities throughout, attempt to bring those displaying more apathy in by gently encouraging them directly, but do so without overstimulating;
- Try to include a diversity of songs to meet all musical interests/cultural backgrounds of the group, but do not be afraid to use the same songs from session to session;
- Songs chosen may be on a theme such as Christmas, School, Love etc. If applicable, add a national theme to these examples to promote dialogue between participants.

3. Improvisation on instruments

This activity is optional from session 2. The MT should revisit/trial these activities at different stages of the group process. Participants who are initially not interested in playing instruments may be more inclined to do so when they know each other and the therapist better and have established a sense of group.

- Provide small hand-held (percussion or small rhythm) instruments to participants;
- Take care to provide light instruments to those who are more frail (e.g., egg shakers, castanets, clave, hand drums, bells);
- Check each participant knows how to play the instrument so they can make an aesthetically pleasing sound when you hand it to them (may be traditional playing sound or adapted for individual needs);
- Select songs or play music in popular accompaniment styles suitable to the musical interests of the group;
- Play and encourage participants to play along with you by moving into a position so you can have eye contact with participants using gesture, modelling and facial expression to motivate participation;
- Extend the song if you can tell the participants are highly engaged in the performance by repeating verses and choruses etc.;
- Encourage participants to play short solos on their instruments if appropriate;
- Encourage singing and playing at the same time if appropriate.

4. Spontaneous or directed movement to music

This activity is optional from session 2. The MT should revisit/trial these activities at different stages of the group process. Participants who are initially not interested or engaged in movement to music may be more inclined to do so when they know each other and the therapist better and have established a sense of group.

- Select 1-2 songs that have an upbeat tempo but are in keeping with the musical preferences and physical abilities of the group;
- Model/encourage either spontaneous OR directed movement to music based on the abilities and interests of participants;
- Spontaneous movement to music:
  - Initiate/model spontaneous movement to music or respond to/mirror participants' spontaneous movements (e.g. tapping feet, marching or "dancing" in seat, swaying, clapping, clicking, "conducting", etc.);
  - Encourage movement both verbally and non-verbally.
- Directed movement to music:
  - Select preferred songs with associated dance movements and model/encourage active participation, including local popular music specific for the region or country;
  - Directed movement to music through specific exercises. Be sure to demonstrate and repeat the movements intermittently to encourage active participation. Examples of specific exercises:
    - Start with gentle breathing awareness;
    - For torso and head: swaying body from side to side, turning body to face your neighbour and then the other way, gently tipping head to left and right shoulder.
    - For upper body/limbs: Rolling shoulders gently backwards, straightening arms out in front and back in, bending elbows (bicep curl action), rotating wrists, opening and closing fingers, lifting one arm above the head and then the other.
    - For lower limbs (while seated): rolling feet backward and forwards (toes to heels), lifting each leg in marching style, lifting lower leg (straightening each leg) +/- rolling ankles.

5. Goodbye song and farewell

- Finish the session by thanking everyone for participating and singing a final goodbye/concluding song to signal the end of the session.
- Choose a goodbye song suitable for the group and use this same song each week as a ritual closure song;
- Remind participants when they will be seeing you again e.g. when the next session will be;
- Tell care staff that attended the session or who pick up/drop off participants when the next session will be (day and time).

Role of Music Therapist facilitators

To provide a variety of targeted musical experiences to support the psychosocial needs of PWD:

- the need for emotion regulation,
- experiencing connections with others,
- feeling validated,
- opportunities to recall and re-experience pleasant memories stimulated by familiar and preferred music,
- verbal, non-verbal and musical communication (who you are, connectedness, and here and now).

The role is also to address agitation and other behavioural symptoms that affect the wellbeing of the PWD and others around him/her.

Plan weekly sessions including development of special themes such as Christmas, Halloween etc.

Ensure there is a spread of songs appropriate to all members of the group. Use a mix of songs for energising but also calming as needed during different parts of the session.

Select songs that match participant preferences and help them validate self identity - "who you are".

Ensure songs are in a comfortable key, volume and pace for the group.

Use eye contact, body gesture and vocal quality to encourage maximum participation from participants with dementia. Move between participants to promote engagement.

Work flexibly to respond to here and now responses and extend responses when they emerge.

Prepare any materials required, for example, selecting recorded music for exercises, creating large print lyrics.

Document attendance and other requirements as outlined in the supporting documents.

Facilitate group cohesion by encouraging participants to be aware of each other (drawing attention to others in the group through musical engagement and verbal dialogue).

Support the social interaction component of the programme.

Use dialogue to support autobiographical recall. For example "X, I understand that this was a song that you used to listen to with Y", "what does that song remind you of?".

When participants are presenting as apathetic or sleepy, using gentle touch to arouse them, move right in front of them and move into a position (perhaps crouching or bending down) so you can gain eye contact, use exaggerated facial expressions but also respect that they might need a little break.

Problem solving when unexpected events occur.

Professional Supervision and Self-Assessment

It is imperative that high quality sessions are provided. This is generating the evidence we want that has the potential to influence policy with respect to funding in the aged care sector. To ensure your sessions are of the highest quality:

Jodie Bloska will facilitate monthly group supervision sessions via Zoom to collaboratively review your sessions by watching video excerpts.

You will review audio-visual recordings of your sessions and complete fidelity checklists. Fidelity checklists are an essential component of our clinical trial because we need to be able to verify that interventions were more or less equivalent irrespective of who the interventionist was, and that the protocol was followed. We will need to report on that in the write up of our findings.

Record your fidelity for the first 8 consecutive sessions. At least 3 of these sessions will be reviewed collaboratively with the researcher supervisors

Once-monthly self assessments of fidelity in collaboration with the researcher supervisors as a way of refreshing expectations etc.

GMT Clinical competencies

Please demonstrate how you would start the first session (i.e. introduction of session)

Please demonstrate how you would start a session after a number of sessions (i.e. recap of previous session, outline plans of session)

Please demonstrate how you would introduce and facilitate movement to music

Please demonstrate how you would facilitate familiar singing and reminiscence

Please demonstrate how you would facilitate instrument playing

Please demonstrate how you would conclude a session

GMT Clinical scenarios – Problem solving

A. You are facilitating a session and a resident is calling out and is becoming disruptive to the group. Please demonstrate how you would manage this situation.

B. You are running a session and you notice that a resident is not engaging in singing/discussion/instrument playing throughout the session. Please show how you would manage this situation.

C. You are conducting a session and a resident asks you that he/she needs to go to the toilet. Please demonstrate how you would deal with this situation.

D. You are in the middle of facilitating a session and a staff member comes into the session to remove a participant for a health check. Please show how you would manage this situation.

E. You are facilitating a session and a family member comes into the session to remove a resident from the group. Please demonstrate how you would respond to this situation.

Test yourself

1. What is the aim of the music therapy assessment and what information do you need to collect?
2. What are the most effective ways of determining the music preferences of the participants?
3. Which research team members need to be blinded to condition? Who can you not tell which home/ward you are providing services to?
4. What are the three principle/theoretical components that should be embedded in the session protocol?
5. What are the 5 main sections of the session format?
6. What therapist techniques can you use to support participants' responses?
7. How would you go about encouraging interaction between participants?
8. What kinds of dialogue would you use to support reminiscence?
9. Name 4 gentle exercises to music that would cover neck/head/torso, arms, legs
10. What music is best suited for gentle exercises to music?
11. What types of music are best suited for use in instrumental improvisation?
12. What would you do if a participant was not engaging with his/her instrument?
13. How might you engage someone who is presenting as apathetic?
14. What would you do if a participant becomes agitated/screams?
15. What would you do if a participant's relative unexpectedly came to visit?
16. What documentation needs to be recorded before the leaving the site?
17. When should you call to remind the residential care site that you are coming?
18. Who should you contact from the research team if you are having issues with compliance from the site?
19. What do you do when a staff member interrupts your group?
20. Why is reviewing your video footage and completing a self-assessment important?

Appendix 1: Music preference form and additional questionnaire for family members

Music Preference Form

Participant name/number:

Date of Session:

Note any personal strengths, resources, disabilities/impairments, cultural background, history*:

Names of songs/music/artists (sourced from resident or family/next of kin):

Songs presented to participant and his/her responses noted: e.g. facial expression, eye contact, verbal communication, singing along, moving to music, postural changes, changes in arousal, agitation etc.:

** If possible, the music therapist will request information in advance about the resident's (musical) background. It is expected that care staff can provide relevant information from patient files and that family also has information about where the resident was born, last school attended, religion etc. Other details about the resident that may be relevant to build the rapport are: the main occupation, names of children and partner, spouse's main occupation, their last job, when and why retired, favourite leisure activity. Care staff or family members may be able to provide these details. These topics may also be useful for entering into a conversation with the resident.*

Music Preference Questionnaire for family members

This questionnaire is based on the evidence-based guideline *Individualized music for elders with dementia* (Gerdner, 2010) and is used for the *Music pillow intervention* by Annemieke Raven-de Vries. You can ask a representative/family member of the participant to fill out this questionnaire prior to the individual assessment to gain insight into the participant's music preferences.

Participant name:……………………………………………………………………………….<br>CHU name:………………………………………………………………………………….<br>Filled out by:……………………………………………………………………………….<br>Relationship to patient:…………………………………………………………………<br>Date:…………………………………………………………………………………………….
How important was music in the life of your next of kin before he / she became ill?<br><br>☐ Very important ☐ Important ☐ Somewhat important ☐ Not important
As far as you can estimate, has the music preference of your next of kin changed due to the illness?<br><br>☐ Not changed ☐ Changed a little bit ☐ Changed ☐ Drastically changed ☐ I don't know<br><br>If changed, please describe briefly:<br>………<br>………
Did he or she play an instrument? ☐ Yes ☐ No ☐ I don't know<br><br>If so, which instrument? (for example: organ, flute)<br>………
Does/Did he or she enjoy singing? ☐ Yes ☐ No ☐ I don't know<br><br>If so, at what times? (for example: during the dishes, in the choir)<br>………
Does/Did he or she enjoy dancing? ☐ Yes ☐ No ☐ I don't know<br><br>If so, please specify (examples: dance lessons, night out)<br>………

Below is a list of different music styles. Can you choose the top 3 of your next of kin?
Write 1 for the most favourite, 2 for the next favourite etc.

☐ Old school songs	☐ Children's songs	☐ Classical music, for example [describe type] …………………………………………………………………………
☐ Rock & Roll	☐ Country/western	☐ Church music, from [indicate church movement] …………………………………………………………………………
☐ Jazz	☐ Blues	☐ Local folk music, such as …………………………………………………………………………
☐ Dutch	☐ March music	☐ Specific cultural music, namely …………………………………………………………………………
☐ Pop music	☐ I don't know	☐ Other, namely …………………………………………………………………………

Can you name specific favorite songs or pieces of music?
Think of specific moments; e.g. which lullabies were sung? Was there music that belonged to holidays? Was there singing in the car? Are there specific funeral songs? Did you family member ever sing something to small children? Etc.
……
……
……
……
……
……
……
……
…………………………………………………………………………

Do you know which artist (s) your next of kin preferred to listen to?
……
……

What are your next of kin's favourite CDs, albums or LPs? ………
………

Appendix 2: Attendance Register to complete at every session

**MIDDEL ATTENDANCE REGISTER**		**DATE:**
**LOCATION:**		**INTERVENTIONIST:**
**START TIME:**		**FINISH TIME:**
**NAME**	**Present Y/N/ Partly**	**IF N GIVE REASON FOR NON-ATTENDANCE, IF PARTLY, WHEN & WHY**

Appendix 3: Covid precautions form to complete at every session

COVID-19

Question:	Yes/No:	
Are any residents or staff currently infected with COVID-19?	☐ **Yes**<br>☐ **No**	**If yes, how many?**<br>Number of residents: …<br>Number of staff: …
Are there currently any COVID-related restrictions?	☐ **Yes**<br>☐ **No**	**If yes, select which applies:**<br>☐ Complete lockdown<br>☐ No access for interventionists<br>☐ No access for visitors<br>☐ Other restrictions, namely<br>…
In case of a lockdown or restricted access, how did you provide the session?		☐ Virtual/online session<br>☐ Session (from) outside<br>…
Are there currently any COVID-related measures in the music sessions?	☐ **Yes**<br>☐ **No**	**If yes, select which applies:**<br>☐ Face masks for participants/ interventionist<br>☐ Screens between participants/interventionist<br>☐ Social distance<br>☐ Smaller groups than usual<br>☐ Only groups from one unit/living room<br>☐ Other restrictions, namely<br>…

Appendix 4: GMT Fidelity Scale

Site code/Group number/Session number:

Date of Session: Session start time: Session end time:

Name of Fidelity Assessor:

Instructions: *check the boxes of sections addressed during the session.*

☐ Session Introduction

☐ Group singing
☐ Improvisation
☐ Movement
☐ Group singing

☐ Session Closure

Instructions: *Score each item as done or not done.*

**Session Introduction (5 minutes):**	☐ **Yes** ☐ **No**	**Comment**
MT uses consistent song to begin session	☐ **Done** ☐ **Not Done**	
MT recaps previous sessions activities	☐ **Done** ☐ **Not Done**	
MT outlines plans for the session	☐ **Done** ☐ **Not Done**	
MT records attendance and reason for non-attendance	☐ **Done** ☐ **Not Done**	
**Session Activity 1: Singing familiar songs (15 minutes)**	☐ **Yes** ☐ **No**	
MT engages participants in singing familiar/preferred songs	☐ **Done** ☐ **Not Done**	
MT facilitates song choice: moves from open- to close-ended choices as needed	☐ **Done** ☐ **Not Done**	
MT facilitates discussion/ reminiscence on at least one occasion in the session	☐ **Done** ☐ **Not Done**	
MT regularly engages each participant individually using eye contact, facial expression and gesture to encourage response	☐ **Done** ☐ **Not Done**	
MT acknowledges and mirrors or reflects participants' spontaneous verbal and non-verbal responses (e.g. singing, vocalizing, moving)	☐ **Done** ☐ **Not Done**	

MT adapts music (extends songs where participants appear highly engaged, adapts tempo, volume, style to attune to overall group energy)	☐ **Done** ☐ **Not Done**	
MT adapts music (as above) to encourage participation from participants displaying apathy or agitation	☐ **Done** ☐ **Not Done** **N/A**	
MT uses appropriate facial expression/adapts gesture, and moves towards participants to encourage individuals and draw out responses	☐ **Done** ☐ **Not Done**	
MT uses a diversity of songs to meet musical interests/cultural background of the group	☐ **Done** ☐ **Not Done**	
**Session Activity 2: Instrument playing (5-7 minutes) - OPTIONAL**	☐ **Yes** ☐ **No** **If no skip 5 rows**	
MT offers choices to participants of instruments to be played – first open-choices and if necessary then closed choices	☐ **Done** ☐ **Not Done**	
MT demonstrates how instruments are to be played and checks each participant knows how to play their instrument by asking participant to demonstrate the playing of the instrument.	☐ **Done** ☐ **Not Done**	
MT verbally and with gesture encourages participants to play along	☐ **Done** ☐ **Not Done**	
MT extends the duration of the song if participants are highly engaged in the performance of a song	☐ **Done** ☐ **Not Done**	
If appropriate, MT encourages participants to play short solos on their instruments	☐ **Done** ☐ **Not Done**	
**Session Activity 3: Spontaneous or directed movement to music (5-7 minutes) - OPTIONAL**	☐ **Yes** ☐ **No** **If no skip 6 rows**	
MT facilitates either spontaneous OR directed movement to music	☐ **Done** ☐ **Not Done**	
MT models movements and encourages participants to move to the music both verbally/non-verbally	☐ **Done** ☐ **Not Done**	
MT spontaneous movement: initiates/models movement to music and/or responds to/mirrors participants' spontaneous movements to music	☐ **Done** ☐ **Not Done**	

Directed movement: MT directs & models specific movements to music (e.g. dances associated with music/songs, specific exercises for head/neck, torso, arms, legs)	☐ **Done** ☐ **Not Done**	
Movements are appropriate for participants' physical abilities, interests and attention spans	☐ **Done** ☐ **Not Done**	
Selected songs are upbeat in tempo and in keeping with participants' musical preferences and physical abilities	☐ **Done** ☐ **Not Done**	
**Session Activity 4: Singing familiar songs (6-10 minutes) – See Session Activity 1**	☐ **Yes** ☐ **No**	
**Session Closure:**	☐ **Yes** ☐ **No**	
Uses consistent song to conclude each session	☐ **Done** ☐ **Not Done**	

**COMMENTS:**

Contacts

Gabrielle Norman, Chiltern Music Therapy

gabrielle@chilternmusictherapy.co.uk

Victoria Crouch, Chiltern Music Therapy

Victoria@chilternmusictherapy.co.uk 01442 780541

Joanne Ablewhite, MIDDEL Trial Manager Nottingham,

Joanne.Ablewhite@nottingham.ac.uk 07929 709853

Justine Schneider, Principal Investigator

Justine.Schneider@nottingham.ac.uk (anytime) 07843 607085 (Wednesdays & Fridays)

@MIDDELProject

MIDDELProject
