# Supplementary material for: Impact of Music Interventions on Depression in Care Home Residents with Dementia: UK Results from Music Interventions for Depression and Dementia in Elderly Care RCT
Source: Geriatrics (Basel). 2025 Dec 15;10(6):166. doi: 10.3390/geriatrics10060166 (PMC12732711; doi:10.3390/geriatrics10060166)
Supplement: Supplementary file 1 [file geriatrics-10-00166-s001.zip › UK RCS Handbook.pdf]

MIDDEL
Music Interventions for Dementia and Depression in Elderly Care

Recreational Choir Singing Groups: Intervention Handbook

December 1, 2021 Version 1.0

Authors

Professor Felicity Baker, University of Melbourne: felicity.baker@unimelb.edu.au

Professor Christian Gold, UniResearch. Bergen, Norway

Dr Jeanette Tamplin, University of Melbourne

Dr Imogen Clark, University of Melbourne

Dr Claire Lee, University of Melbourne

Funded by the National Health and Medical Research Council, Australia

Adapted by Justine Schneider for UK MIDDEL study

Contents

Recreational Choir Singing Groups: Intervention Handbook 1
Authors 1
Overview of intervention 2
Group size 3
Essential skills for group leader 3
Key responsibilities 3
Key features of the intervention 4
Theoretical underpinnings 4
Aims of RCS 4
Important considerations 5
Setting up group sessions: preparation 5
Tasks to be completed prior to each session: 5
Considerations regarding health & safety 6
After the session 6
Session outline 7
1. Introduction 7
2. Physical and Vocal Warm-up and Exercises 7
3. Singing familiar songs 7
4. Learning new songs 8
5. Goodbye song and farewell 8
Song materials 8
Role of choir leaders 8
Covid precautions form to complete at each session 10
Attendance register to complete at every session 11
Facilitator Fidelity Checklist RCS 12

Overview of intervention

Group size

10-20 participants (minimum 10)

- Weeks 1-13, 2 x 45 minute sessions
- Weeks 14-26, 1 x 45 minute sessions per week

Essential skills for group leader

- Experience in conducting amateur choirs for adults
- Capacity to accompany the choir on either keyboard or guitar
- Sound written and communication skills including the ability to communicate with a range of stakeholders
- Sound computer and internet skills
- Demonstrated ability to work independently and collaboratively in a team to achieve goals and meet deadlines
- Strong organisational skills

Key responsibilities

- Attend choir training sessions as provided by the research team
- Conduct group singing/choir directing with groups of people recruited to the study in various aged care facilities at the scheduled times
- Keep a log of attendance of participants
- Work collaboratively with care home staff to ensure sessions commence and conclude on schedule
- Attend teleconference team meetings as scheduled by the research team
- Report regularly to Gabrielle Norman and to Joanne Ablewhite, who will have oversight of the project

Key features of the intervention

- Creative process together where everyone has a meaningful contribution to the group
- ”Produce” music together by singing
- Relatively structured, goal-oriented, cognitive activation
- Relationship between conductor and group (as a whole)
- ”Recreational” = positive, fun and but relatively structured.
- Repertoire: mix of new and familiar
- Supporting social processes

Theoretical underpinnings

- Combination of cognitive, physical and psychosocial engagement
- Leisure-based, singing for enjoyment
- Promote social interaction and connection
- Stimulation of mesolimbic system (pleasure centre)
- Activation of positive emotions

Aims of RCS

- Warm up routine (for vocal hygiene as well as warm up to assist with singing)
- Singing (enjoyment and skill)
- Stimulation of autobiographical memories
- Opportunities for conversation
- Opportunities for active participation
- Opportunities for social connection with others

Important considerations

1. Each session runs for approximately 45 minutes and will be video recorded.
2. Wherever possible, if sessions are missed due to illness, they should be made up. (Note: You will be prompted by the trial manager Joanne Ablewhite when the sessions will reduce to 1 session per week).
3. Please inform Joanne as early as possible if you are going to miss a session due to illness or other significant events.

Setting up group sessions: preparation

- Learn repertoire that is identified as being familiar from the era when participants were in their 20s (reminiscence bump)
  - for participants who are 65-75, music from 50s and 60s,
  - for participants who are 75+ music from 30s and 40s.
  - Common songs such as football songs, National Anthem, Christmas Carols, some church music such as Ave Maria etc and music from musicals/films (Wizard of Oz, Singing in the Rain, Oliver, My Fair Lady, The Sound of Music, Mary Poppins etc)
- Song selection – sourcing of sheet music / chord charts as required
- Environment and equipment suggestions
- Large room for 20-30 people
- Piano or good quality keyboard with functions for transposing, rhythmic loops, changing sounds and/or guitar and stand
- Device for uploading videos – must run Chrome or Firefox should be charged in advance

Tasks to be completed prior to each session:

- Reminder/confirmation of visit the morning of each session (to be arranged with nominated person at the home) – text/phone call and email.
- Arrive at the venue early enough to allow set-up outside the 45 minute delivery time.
- Set up recording device and position it, log on to University website and start recording
- In collaboration with care home staff, set up chairs and place large print lyrics on chairs and/or power point projectors
- Prepare attendance list for each session

Considerations regarding health & safety

Environmental factors can impact significantly on the ability of people with dementia to function optimally. In setting up for RCS groups, consider:

Need for wide, open spaces. Narrow spaces can increase the risk of falls. Ensure that walking areas and paths created when setting up chairs are wide and clear of obstacles.

Clutter can impact on mobility and safety. Reduce clutter and obstacles to facilitate mobility.

Some people with dementia may have difficulty with chair transfers (getting in and out of a chair). Chairs with arms can assist with transfers. Nursing and personal care staff can assist. Please always ask for help when needed.

Allow participants to sit or stand whilst singing, depending on their balance and postural stability. Suggest that if they would like to stand but feel unstable, they can stand behind a chair and use it for support. Encourage sitting if balance and postural stability are particularly poor or if fatigue is an issue.

Be aware of the mobility needs of each participant, especially if there are specific strategies or aids and equipment recommended.

After the session

- Stop and label the recording
- Pack up and assist where necessary to take participants back to rooms or general areas
- Check completion of session documentation
- Check in with Joanne to see if all documentation has been complete and that audio-visual files have been synced to the cloud
- Report any issues or untoward events to Joanne and Gabrielle

Session outline

1. Introduction

- Welcome song (choose a song and use at the beginning of each session) for example, Hello, Hello, Whose Your Lady Friend; Oh What A Beautiful Morning (if morning session), You Are My Sunshine;
- Recap of previous session or current events
- Outline of plan for the session

2. Physical and Vocal Warm-up and Exercises

- 3-4 minutes of vocal warm-up and exercises.
- Physical warmups including gentle stretching and posture awareness exercises
- Vocal warm up including exercises to support breathing, pitch range, rhythmic awareness, vocal agility, dynamics, and harmony.
- This will include a focus on each of the following areas:
    - Posture
    - Breathing
    - Humming/vowel prolongations
    - Scales and glides
    - Arpeggios and thirds scales
    - Diction practise/tongue twisters
    - Familiar song singing

3. Singing familiar songs

- Present an index of songs on large print sheets and encourage participants to choose songs from the list. Present lyrics in large print and/or via projector
- Ask for song suggestions and genre preferences
- Aim for at least 30 minutes of singing participant-selected songs
- Use a prepared list of songs, try to re-use the same songs from week to week
- Songs within a comfortable vocal range with adequate pitch variation are desirable

4. Learning new songs

- Aim for opportunity for participants to learn new songs, or harmony parts to familiar songs.
- Some songs will need to be taught gradually in sections or parts
- Rounds are a good way of introducing harmony
- Try mash ups (for example, combination of "When the Saints", "Sing-Sing-Sing", "Swing Low").

5. Goodbye song and farewell

- Finish the session by thanking everyone for participating and singing a final goodbye/ concluding song to signal the end of the session.

Song materials

- We can supply songbooks compiled for the PRESIDE study (50 songs)

Role of choir leaders

- Ensure group input into song choices
- Use a mix of familiar and novel songs
- Prepare a song index and lyrics to help with selection, good singing posture, and engagement.
- Provide musical accompaniment to vocal exercises and songs
- Prepare musical activities, selecting keys, modifying song structures, etc
- Ensure songs are in a comfortable key and pace for the group
- Seek feedback and input from participants about music preferences and group activities
- Prepare any materials required, for example, recording and copying of CD's to be used for vocal exercise and singing practice between group sessions

Covid precautions form to complete at each session

**Question:**	**Yes/No:**	
Are any residents or staff currently infected with COVID-19?	☐ **Yes**<br>☐ **No**	**If yes, how many?**<br>Number of residents: …<br>Number of staff: …
Are there currently any COVID-related restrictions?	☐ **Yes**<br>☐ **No**	**If yes, select which applies:**<br>☐ Complete lockdown<br>☐ No access for interventionists<br>☐ No access for visitors<br>☐ Other restrictions, namely<br>…
In case of a lockdown or restricted access, how did you provide the session?		☐ Virtual/online session<br>☐ Session (from) outside<br>…
Are there currently any COVID-related measures in the music sessions?	☐ **Yes**<br>☐ **No**	**If yes, select which applies:**<br>☐ Face masks for participants/ interventionist<br>☐ Screens between participants/interventionist<br>☐ Social distance<br>☐ Smaller groups than usual<br>☐ Only groups from one unit/living room<br>☐ Other restrictions, namely<br>…

Attendance register to complete at every session

MIDDEL ATTENDANCE REGISTER		DATE:
LOCATION:		INTERVENTIONIST:
START TIME:		FINISH TIME:
NAME	Present Y/N/ Partly	IF N GIVE REASON FOR NON-ATTENDANCE, IF PARTLY, WHEN & WHY

Facilitator Fidelity Checklist RCS

Site code/Session number: Date of Session:

Session start time: Session end time:

Name of Fidelity Assessor:

Instructions: Score each item as done or not done or yes or no as indicated in the form.

**Session Introduction:**	**Circle one**	**Comment**
Facilitator uses consistent song to begin session (welcome song)	Done/ Not Done	
Facilitator recaps previous sessions activities	Done/ Not Done	
Facilitator outlines plans for the session	Done/ Not Done	
Facilitator records attendance and reason for non-attendance (eg. Sick)	Done/ Not Done	
**Session Activities:**		
Facilitator provides simple physical warm up: breathing, stretching, posture awareness (3-4 mins)	Done/ Not Done	
Facilitator provides simple vocal warm up: breathing, stretching, posture awareness: vocal agility exercises, dynamics, harmony, humming/vowel prolongations, scales/glides/diction, arpeggios and 3rds scales (3-4 mins)	Done/ Not Done	
Facilitator engages participants in singing participant-selected songs for 30 mins	Done/ Not Done	
Songs are performed with comfortable range	Done/ Not Done	
Facilitator provides lyrics in large print	Done/ Not Done	
**Session Closure:**		
Facilitator used consistent song to conclude each session	Done/ Not Done	
Number of fidelity criteria met		

Comment overleaf on any extenuating circumstances or untoward events.

@MIDDELProject

MIDDELProject
